# High *EVI1* Expression Predicts Adverse Outcomes in Children With *De Novo* Acute Myeloid Leukemia

**DOI:** 10.3389/fonc.2021.712747

**Published:** 2021-09-13

**Authors:** Yongzhi Zheng, Yan Huang, Shaohua Le, Hao Zheng, Xueling Hua, Zaisheng Chen, Xiaoqin Feng, Chunfu Li, Mincui Zheng, Honggui Xu, Yingyi He, Xiangling He, Jian Li, Jianda Hu

**Affiliations:** ^1^Department of Hematology, Fujian Institute of Hematology, Fujian Provincial Key Laboratory on Hematology, Fujian Medical University Union Hospital, Fuzhou, China; ^2^Department of Pediatrics, Southern Medical University/Nanfang Hospital, Guangzhou, China; ^3^Nanfang-Chunfu Children’s Institute of Hematology & Oncology, TaiXin Hospital, Dongguan, China; ^4^Hematology and Oncology, Hunan Children’s Hospital, Changsha, China; ^5^Department of Pediatric Hematology & Oncology, Sun Yat-sen Memorial Hospital, Guangzhou, China; ^6^Department of Pediatric Hematology/Oncology, Guangzhou Women and Children’s Medical Center, Guangzhou, China; ^7^Pediatrics, People’s Hospital of Hunan Province, Changsha, China

**Keywords:** *EVI1*, prognostic factor, acute myeloid leukemia, pediatric, adverse outcome, transplantation

## Abstract

**Background:**

A high ecotropic viral integration site 1 (*EVI1)* expression (*EVI1* ^high^) is an independent prognostic factor in adult acute myeloid leukemia (AML). However, little is known of the prognostic value of *EVI1* ^high^ in pediatric AML. This study aimed to examine the biological and prognostic significance of *EVI1* ^high^ in uniformly treated pediatric patients with AML from a large cohort of seven centers in China.

**Methods:**

A diagnostic assay was developed to determine the relative *EVI1* expression using a single real-time quantitative polymerase chain reaction in 421 newly diagnosed pediatric AML patients younger than 14 years from seven centers in southern China. All patients were treated with a uniform protocol, but only 383 patients were evaluated for their treatment response. The survival data were included in the subsequent analysis (n = 35 for *EVI1* ^high^, n = 348 for *EVI1* ^low^).

**Results:**

*EVI1*^high^ was found in 9.0% of all 421 pediatric patients with *de novo* AML. *EVI1* ^high^ was predominantly found in acute megakaryoblastic leukemia (FAB M7), *MLL* rearrangements, and unfavorable cytogenetic aberrance, whereas it was mutually exclusive with t (8; 21), inv (16)/t (16; 16), *CEBPA*, *NPM1*, or *C-KIT* mutations. In the univariate Cox regression analysis, *EVI1* ^high^ had a significantly adverse 5-year event-free survival (EFS) and overall survival (OS) [hazard ratio (HR) = 1.821 and 2.401, *p* = 0.036 and 0.005, respectively]. In the multivariate Cox regression analysis, *EVI1* ^high^ was an independent prognostic factor for the OS (HR = 2.447, *p* = 0.015) but not EFS (HR = 1.556, p = 0.174). Furthermore, *EVI1* ^high^ was an independent adverse predictor of the OS and EFS of patients with *MLL* rearrangements (univariate analysis: HR = 9.921 and 7.253, both *p* < 0.001; multivariate analysis: HR = 7.186 and 7.315, *p* = 0.005 and 0.001, respectively). Hematopoietic stem cell transplantation (HSCT) in first complete remission (CR1) provided *EVI1* ^high^ patients with a tendential survival benefit when compared with chemotherapy as a consolidation (5-year EFS: 68.4% *vs.* 50.8%, *p* = 0.26; 5-year OS: 65.9% *vs.* 54.8%, *p* = 0.45).

**Conclusion:**

It could be concluded that *EVI1* ^high^ can be detected in approximately 10% of pediatric AML cases. It is predominantly present in unfavorable cytogenetic subtypes and predicts adverse outcomes. Whether pediatric patients with *EVI1* ^high^ AML can benefit from HSCT in CR1 needs to be researched further.

## 1 Introduction

Acute myeloid leukemia (AML) accounts for approximately 20% of pediatric leukemia diagnoses, and its long-term survival rate has dramatically increased from less than 20% to approximately 70% in the past 50 years (1). One of the most important reasons for these dramatic improvements is the accurate prediction of the prognosis to initiate the appropriate therapy regimens. Cytogenetic abnormalities and gene mutations are the classical and most important framework for risk stratification (2, 3). In addition to genetic alterations, aberrant overexpression of specific genes may also serve as biomarkers to evaluate the risk of treatment failure or relapse; ecotropic viral integration site 1 (*EVI1*) is a representative of this group (4, 5).

The *EVI1* gene encodes a zinc-finger protein that functions as a transcription factor essential for hematopoietic stem cell (HSC) proliferation and differentiation, and is located on chromosome 3q26 (6). Aberrantly high *EVI1* expression (*EVI1* ^high^) plays an important role in the pathogenesis of hematological malignancies, including AML, chronic myeloid leukemia, and myelodysplastic syndrome (MDS) (7). In adult AML, *EVI1* ^high^ is frequently associated with cytogenetic abnormalities of 3q, especially 3q26, whereas in pediatric AML, it is rarely correlated with the cytogenetic rearrangements of this locus (8). In pediatric AML, *EVI1* ^high^ is commonly found together with mixed lineage leukemia (*MLL*) rearrangements, which indicates that the pathogenetic and prognostic significance of *EVI1* ^high^ may be different between adult and pediatric patients with AML (9, 10).

In the past decade, several studies have shown that *EVI1* ^high^ is a poor independent prognostic predictor for the complete remission (CR), overall survival (OS), relapse-free (RFS), and event-free survival (EFS) in adult AML, irrespective of the cytogenetic abnormalities of 3q (8). However, few studies have examined how *EVI1* ^high^ affects the prognosis in pediatric AML, and some of its effects are contradictory to those of adult AML. Using multivariate analysis, Balgobind et al. (10) and Ho et al. (9) reported that *EVI1* ^high^ had a significantly lower EFS but was not independently associated with an inferior OS and EFS in pediatric AML. In consideration of the inconsistent conclusions, the aim of our study was to examine the biological and prognostic significance of *EVI1* ^high^ in uniformly treated pediatric AML patients from a large cohort of seven centers in China.

## 2 Material and Methods

### 2.1 Patients and Treatment

A total of 421 newly diagnosed pediatric patients with AML (≤14 years) were enrolled in this retrospective study. Patients with acute promyelocytic leukemia, secondary AML, constitutional trisomy 21, or antecedent MDS were excluded. These patients were consecutively diagnosed at seven centers in southern China between January 2015 and December 2020. Morphological, flow cytometric, cytogenetic, and molecular analyses were performed on all patients at diagnosis, and the results were available for all patients included in this study. AML was diagnosed and classified according to the World Health Organization (2016) classification (11).

All patients were treated using the C-HUANAN-AML15 protocol. In the C-HUANAN-AML15 protocol, two tandem courses of the FLAG-IDA or DAE regimen were applied as induction chemotherapy. One course of homoharringtonine cytarabine/etoposide and one course of mitoxantrone/cytarabine in consolidation chemotherapy were uniformly administered to both groups. Intermediate-risk patients who had human leukocyte antigen (HLA) matched donors and high-risk patients were advised to undergo HSC transplantation (HSCT) in CR1. Details of the treatment protocols are provided in the **Supplementary Data** section. This study was approved by the ethics committee of all seven centers. All patients and volunteers provided a written informed consent, in accordance with the Declaration of Helsinki, to participate in the present study.

### 2.2 Quantitative Real-Time Polymerase Chain Reaction (qRT-PCR)

Total RNA was isolated from nucleated cells of the bone marrow using the Trizol Reagent (Invitrogen, Carlsbad, CA, USA), and subsequent reverse transcription was performed on 1 μg of the total diagnostic RNA using the standard protocol (Invitrogen, Carlsbad, CA, USA). *EVI1* expression was measured by qRT-PCR using the TaqMan Universal PCR Master Mix and TaqMan *EVI1* Gene Expression Assay (Applied Biosystems, Foster City, CA) with a primer/probe set designed to hybridize within a region spanning exons 2 and 3, as follows:

Forward primer: 5’-GTACTTGAGCCAGCTTCCAACA-3’ (in exon 3)
Reverse primer: 5’-CTTCTTGACTAAAGCCCTTGGA-3’ (in exon 2)
Probe: 5’-FAM-TCTTAGACGAATTTTACAATGTGAAGTTCTGCATAGATG-TAMRA-3’ (in exon 3).

Abelson murine leukemia viral oncogene homolog *(ABL)* was used as a control gene, and the corresponding primers and probes were based on a report from the Europe Against Cancer Program (12). The primer of *EVI1* can detect the expression of the total *EVI1* (including *EVI1-1A*, *1B*, *1C*, and *1D*), and the *MDS1* and *EVI1* complex fusion transcript. A total of 15 bone marrow samples from healthy donors were processed as calibrators for quantification. *EVI1* transcript levels were calculated as the percentage of the target transcript copies/*ABL* copies. The mean value of *EVI1* in the 15 normal controls was considered as the baseline. Relative quantification was performed using the 2^-△△Ct^ method (13). The relative expression level of *EVI1* is expressed as a percentage. The percentile of the expression level in all *EVI1* test results in the present study was used as a percentage. *EVI1* expression levels were dichotomized based on a cutoff value of 75% (approximately 10 times the baseline), according to Santamaría et al. (14). A total of 38 patients were defined as having *EVI1* ^high^ and the remainder as *EVI1* ^low^.

### 2.3 Statistical Analysis

Continuous variables of patient characteristics were compared using the Wilcoxon rank-sum test (abnormal distribution) or Mann–Whitney U test (normal distribution), while categorical variables were compared using Pearson’s χ^2^ test or Fisher’s exact test when data were sparse. A total of 38 children (3 children with *EVI1* ^high^ and 35 children with *EVI1* ^low^), including those that were lost during the follow-up (n = 21), discontinued the treatment (n = 12), or were transferred (n = 5) before completing two courses of chemotherapy or were excluded from the survival analysis. The cutoff date for follow-up was February 28, 2021. OS endpoints were death (failure) and being alive at the last follow-up (censored), measured from the onset to the start of chemotherapy. EFS endpoints were disease relapse or death from any cause, measured from the onset to the start of chemotherapy. Distribution estimations and survival distributions of the OS and EFS were calculated using the Kaplan-Meier method and log-rank test, respectively. Univariate analyses were performed using the unadjusted Cox proportional hazards model to calculate the hazard ratios (HRs). Variables that were significant in the univariate analyses were included in the multivariate analyses. Multivariate analyses were performed using the Cox proportional hazards model to identify the independent prognostic factors. All tests were two-sided, and a *p*-value of less than 0.05 was considered statistically significant. All statistical analyses were performed using the Statistical Software Environment R, version 4.0.4.

## 3 Results

### 3.1 Clinical Characteristics of Pediatric Patients With Acute Myeloid Leukemia (AML) and a High Ecotropic Viral Integration Site 1 Expression (*EVI1* ^high^)

The clinical features of all 421 patients are summarized in **Table 1**. The median age was 74 months (range, 7–176 months), and 12 patients had infant AML. The most common cytogenetic changes included t (8; 21) and 11q23 chromosome abnormality. In 47/421 (11.2%) patients, FLT3-ITD mutations were detected, while ASXL1 mutations occurred in 11.6%. *EVI1* ^high^, which was found in 9.0% (38/421) of all the patients, was not detected in infant patients. *EVI1* ^high^ was not correlated with age, sex, or white blood cells (WBC), whereas patients with *EVI1* ^high^ had a significantly higher frequency of (1) acute megakaryoblastic leukemia (FAB-M7) (23.7% *vs.* 6.0%, *p* = 0.001) (2), *MLL* rearrangements (39.5% *vs.* 14.4%, *p* < 0.0001), especially *MLL-AF9* (15.8% *vs.* 6.8%, *p* = 0.046) (3); unfavorable cytogenetic aberrance (55.3% *vs.* 24.8%, *p* < 0.0001), but only one patient harbored a 3q26 rearrangement. *EVI1* ^high^ was not found in the favorable subtypes, including t (8;21) and inv (16). We also studied *EVI1* ^high^ in relation to five common single-gene mutations. Three patients with *EVI1* ^high^ had an internal tandem duplication of FMS-like tyrosine kinase 3 (*FLT3-ITD)*, and three patients with *EVI1* ^high^ had an *ASXL1* mutation; this association was not statistically significant compared to patients with *EVI1* ^low^. *EVI1* ^high^ was not found in patients with *NPM1*, *C-KIT*, or *CEBPA*-biallelic mutations.

**Table 1:** Clinical and genetic characteristics according to the *EVI1* status.

Characteristic	All patients (n = 421)	*EVI1*^high^ group (n = 38)	*EVI1*^low^ group (n = 383)	*P-value*
**Age, months**				**0.964**
**Median (range)**	74 (7–176)	44 (12–163)	75 (7–176)	
**Sex, n%**				**0.487**
**Male**	264 (62.7)	26 (68.4)	238 (62.1)	
**Female**	157 (37.3)	12 (31.6)	145 (37.9)	
**WBC, ×10^9^/L**				**0.137**
**<50**	294 (69.8)	31 (81.6)	263 (68.7)	
**≥50**	127 (30.2)	7 (18.4)	120 (31.3)	
**FAB subtype, n%**				**0.001**
**M7**	32 (7.6)	9 (23.7)	23 (6.0)	
**Other types**	389 (92.4)	29 (76.3)	360 (94.0)	
*Cytogenetic characteristics, n (%)				
**t (8;21)**	106 (25.2)	0 (0)	106 (27.7)	**<0.001**
**inv (16)/t (16;16)**	28 (6.7)	0 (0)	28 (7.3)	**0.072**
**t (v;11q23)**	70 (16.6)	15 (39.5)	55 (14.4)	**<0.001**
**t (9;11)**	32 (7.6)	6 (15.8)	26 (6.8)	**0.046**
**-7 or del (7q)**	17 (4.0)	3 (7.9)	14 (3.7)	**0.385**
**Complex karyotype**	13 (3.1)	3 (7.9)	10 (2.6)	**0.390**
**†Cytogenetic risk**				**<0.001**
**Favorable**	128 (30.4)	0 (0)	128 (33.4)	
**Intermediate**	177 (42.0)	17 (44.7)	160 (41.8)	
**Unfavorable**	116 (27.6)	21 (55.3)	95 (24.8)	
**Molecular abnormalities**				
**FLT3-ITD**	47 (11.2)	3 (7.9)	44 (11.5)	**0.602**
**ASXL1**	49 (11.6)	3 (7.9)	46 (12.0)	**0.600**
**CEBPA- mutation**	13 (3.1)	0 (0)	13 (3.4)	**0.309**
**NPM1- mutation**	9 (2.1)	0 (0)	9 (2.3)	**0.462**
**C-KIT-mutation**	40 (9.5)	0 (0)	40 (10.4)	**0.020**
**^#^CR after induction 2^nd^**				**0.364**
**Yes**	342 (81.4)	29 (76.3)	313 (81.9)	
**No**	31 (7.4)	2 (5.3)	29 (7.6)	
**Missing**	47 (11.2)	7 (18.4)	40 (10.5)	
**^#^Blast>15% in BM after induction 1^st^**				**1.000**
**Yes**	20 (4.8)	2 (5.3)	18 (4.7)	
**No**	390 (92.6)	35 (92.1)	355 (92.7)	
**Missing**	11 (2.6)	1 (2.6)	10 (2.6)	

*Patients may be counted more than once owing to the coexistence of more than one cytogenetic abnormality in the leukemic clone.

†Favorable risk: t (15;17), t (8;21), inv (16)/t (16;16); unfavorable risk: inv (3) or t (3;3),t (6;9), t (v;11q23) other than t (9;11), -5 or del (5q), -7 or del (7q), abn (17p), complex karyotype (three or more abnormalities in the absence of a WHO designated recurring chromosome abnormality); intermediate risk: all chromosome abnormalities not classified as favorable or unfavorable. #Only 383 patients were included in this part, for 38 cases giving up treatment or loss to follow-up.

Bold values indicated statistically significant differences.

### 3.2 Survival Analysis of Whole Pediatric Acute Myeloid Leukemia (AML)

An evaluation of the treatment response and the survival data were only available for 383 patients, including 35 patients with *EVI1* ^high^, because some of them discontinued treatment or were lost to follow-up. The median follow-up time for survival was 32.5 (range, 2.6–134.1) months. Of the 383 cases, the CR rate was 85.6% (328/383) and 91.7% (342/373) after the first and second courses of induction, respectively; the relapse rate was 19.5% (64/328) and the chemotherapy-related mortality was 4.7% (18/383). The 5-year EFS and OS were 66.7% and 75.2%, respectively.

### 3.3 Survival Analysis of Pediatric Acute Myeloid Leukemia (AML) With a High Ecotropic Viral Integration Site 1 Expression (*EVI1* ^high^)

For the 35 patients with *EVI1* ^high^, 32 patients (91.4%) achieved CR and 26 (74.3%) patients achieved MRD negative after the first course of induction, whereas 27 patients (93.1%) achieved CR and 25 (86.2%) patients achieved MRD negative after the second course of induction, respectively; the relapse rate was 20% (7/35) and the chemotherapy-related mortality was 2.9% (1/35).

**Figure 1:**
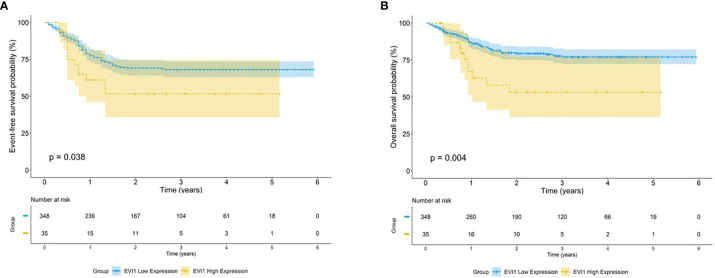
Survival outcome for high EVI1 expression in pediatric AML. Kaplan–Meier curve estimates for the **(A)** EFS and **(B)** OS in the total cohort between *EVI1* ^high^ and *EVI1* ^low^ patients.

The proportion of patients with bone marrow blasts >15% after the first course of induction, and CR rate after the second course of induction was not statistically different between the *EVI1* ^high^ and *EVI1* ^low^ subgroups. Patients with *EVI1* ^high^ had a significantly worse 5-year EFS and OS than those with *EVI1^low^* (EFS: 51.7% *vs.* 68.1%, *p* = 0.041; OS: 53.1% *vs.* 77.0%, *p* = 0.041) (**Figure 1**).

**Figure 2:**
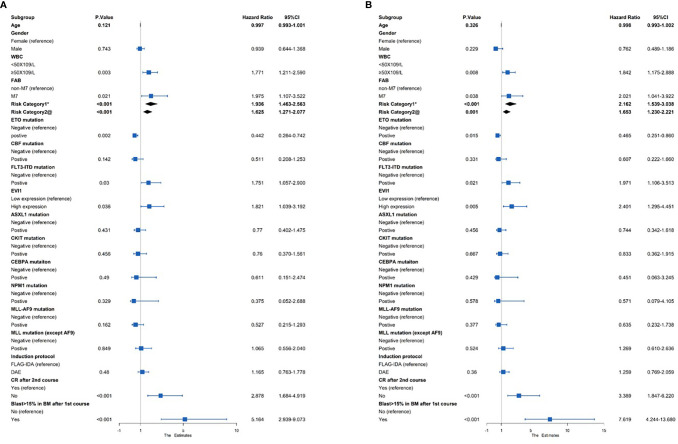
Univariate Cox regression analysis of the **(A)** EFS and **(B)** OS among 383 pediatric AML patients. *Risk category based on treatment regimens. Refer to **Supplementary Table S1**. ^@^Risk category based on cytogenetic stratification of ELN 2017.

Risk factors, including age, sex, WBC, FAB type, risk category, and genetic abnormalities, were evaluated using a univariate Cox analysis (**Figure 2**). *EVI1* ^high^ was a significant factor for the decreased EFS and OS (HR = 1.821 and 2.401, *p* = 0.036 and 0.005, respectively). In addition, poor prognostic predictors also included WBC ≥ 50 × 10^9^/L, risk category, *FLT3-ITD* mutation, failure to achieve CR after the second course of induction, and the proportion of bone marrow blasts higher than 15% after the first course of induction (all HR > 1 and *p* < 0.05). In contrast, an *AML1-ETO* as a favorable predictor of improved the EFS and OS (HR = 0.442 and 0.465, *p* = 0.002 and 0.015, respectively).

**Figure 3:**
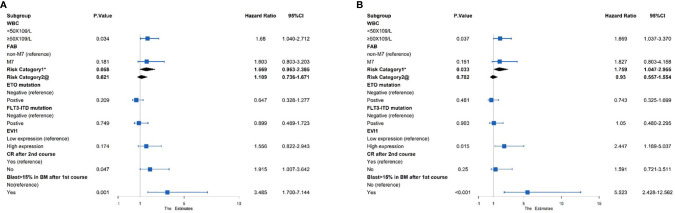
Multivariate Cox regression analysis of the **(A)** EFS and **(B)** OS among 383 pediatric AML patients. *Risk category based on treatment regimens. Refer to **Supplementary Table S1**. ^@^Risk category based on cytogenetic stratification of ELN 2017.

A multivariate Cox analysis was then performed to evaluate the independent prognostic factors (**Figure 3**). The results showed that *EVI1* ^high^ significantly affected the OS (HR = 2.447, *p* = 0.015) but not EFS (HR = 1.556, *p* = 0.174). Similarly, the risk category based on the treatment program was a poor independent prognostic predictor for the OS (HR = 1.759, *p* = 0.033) and tendentially predicted a worse EFS (HR = 1.569, *p* = 0.058). A WBC count ≥ 50 × 10^9^/L and a proportion of bone marrow blasts greater than 15% after the first course were independent risk predictors for the OS and EFS (all HR > 1 and *p* < 0.05). However, failure to achieve CR after two courses of induction treatment was an independent prognostic factor for an inferior EFS (HR = 1.915, *p* = 0.047), excluding the OS (HR = 1.591, *p* = 0.25).

### 3.4 Characteristics and Prognostic Value of High Ecotropic Viral Integration Site 1 Expression (*EVI1* ^high^) in Patients With *MLL* Rearrangements

**Figure 4:**
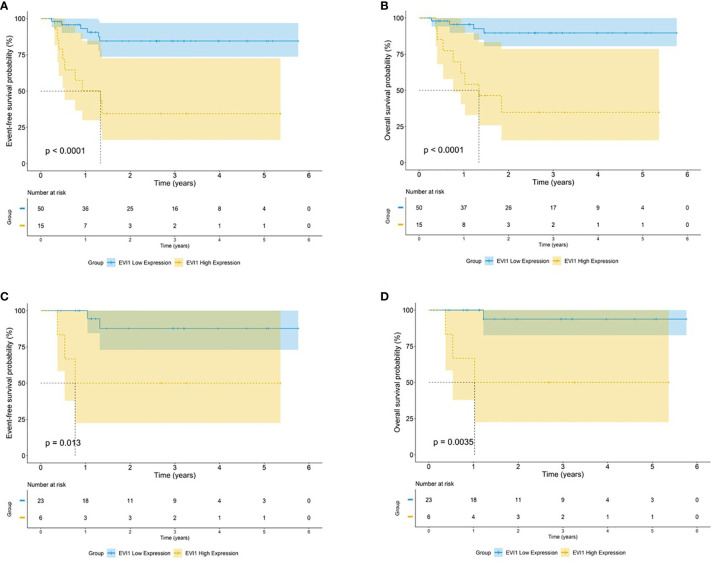
Survival outcomes by *EVI1* expression among patients carrying t(v;11). Kaplan–Meier curve estimates for the **(A)** EFS and **(B)** OS in the cohort of MLL rearranged AML between *EVI1* ^high^ and *EVI1* ^low^ patients. Kaplan–Meier curve estimates for the **(C)** EFS and **(D)** OS in the cohort of *MLL-AF9* rearranged AML between *EVI1* ^high^ and *EVI1* ^low^ patients.

As noted above, *EVI1* ^high^ was associated with *MLL* rearrangement. *EVI1* ^high^ was detected in 21.4% (15/70) of all patients with *MLL* rearrangements and 18.8% (6/32) of patients with *MLL-AF9*. The characteristics of patients with *MLL* rearrangements categorized according to the *EVI1* status are shown in **Supplementary Table S2** *. EVI1^high^* was significantly correlated to an unfavorable cytogenetic aberrance (73.3% *vs.* 38.2%, both *p* < 0.02). *EVI1* ^high^ had a significantly adverse 5-year EFS and OS in all patients with *MLL* rearrangements (EFS: 34.5% *vs.* 84.5%, OS: 34.8% *vs.* 89.7%, both *p* < 0.0001) (**Figure 4**). The same conclusion was found in patients with *MLL-AF9* (EFS: 50.0% *vs.* 87.7%, *p* = 0.013; OS: 50.0% *vs.* 93.7%, *p* = 0.0035) (**Figure 4**).

A Cox regression analysis was performed. Only 65 patients with *MLL* rearrangements were included. The results revealed that *EVI1* ^high^ was an independent adverse predictor of the OS and EFS in patients with *MLL* rearrangements (univariate analysis: HR = 9.921 and 7.253, both *p* < 0.001; multivariate analysis: HR = 7.186 and 7.315, *p* = 0.005 and 0.001, respectively) (**Table 2**). Among the AML patients with *MLL-AF9*, significant differences in the OS and EFS were observed in the univariate analysis (HR = 13.349 and 7.112, *p* = 0.025 and 0.032, respectively); however, multivariate analysis did not identify *EVI1* ^high^ as an independent prognostic factor for the OS and EFS (HR = 13.056 and 10.091, *p* = 0.060 and 0.066, respectively; **Table S2**).

**Table 2:** Univariate and multivariate analysis of patients with *MLL* rearrangement.

Cases (n = 65)	EFS			OS		
Univariate Analysis	*HR*	95% CI	*P*-value	*HR*	95% CI	*P*-value
Age (+1 year)	0.993	0.981–1.006	0.292	0.997	0.984–1.009	0.602
Gender (Male)	0.824	0.299–2.274	0.709	0.706	0.227–2.191	0.547
WBC (≥50X109/L)	2.600	0.943–7.173	0.065	3.115	0.988–9.816	0.052
FAB (M7)	2.333	0.522–10.418	0.267	1.203	0.155–9.348	0.860
Risk Category1*	*2.259*	0.771–6.616	0.137	3.287	1.041–10.381	**0.043**
Risk Category2@	2.599	0.888–7.606	0.081	3.924	1.062–14.504	**0.040**
FLT3-ITD mutation	1.078	0.142–8.210	0.942	1.443	0.186–11.192	0.726
*EVI1* ^high^	7.253	2.559–20.561	**<0.001**	9.921	2.954–33.319	**<0.001**
ASXL1 mutation	0.041	0.000–39.818	0.363	0.041	0.000–97.422	0.421
Induction protocol (DAE)	3.390	1.226–9.371	**0.019**	2.704	0.857–8.528	0.090
No CR after 2nd course	8.280	2.252–30.448	**0.001**	6.891	1.438–33.021	**0.016**
Blast>15% in BM after 1st course	1.962	0.442–8.705	0.376	2.760	0.604–12.618	0.190
**Multivariate Analysis**	**HR**	**95% CI**	***P*-value**	**HR**	**95% CI**	***P*-value**
Risk Category1*	/	/	/	0.985	0.206–4.715	0.985
Risk Category2@	/	/	/	1.593	0.331–7.667	0.561
Induction protocol (DAE)	3.284	0.849–12.707	0.085	/	/	/
*EVI1* ^high^	7.315	2.208–24.229	**0.001**	7.186	1.843–28.019	**0.005**
**No CR after 2nd course**	3.046	0.625**–**14.835	0.168	4.840	0.836**–**28.032	0.078

*Risk category based on treatment regimens. Refer to **Supplementary Table S1**.

^@^Risk category based on cytogenetic stratification of ELN 2017.

Bold values indicated statistically significant differences.

### 3.5 The Impact of a Different Induction Regimen and Effect of Hematopoietic Stem Cell Transplantation (HSCT) after First Complete Remission (CR1) in Patients With a High Ecotropic Viral Integration Site 1 Expression (*EVI1* ^high^)

**Figure 5:**
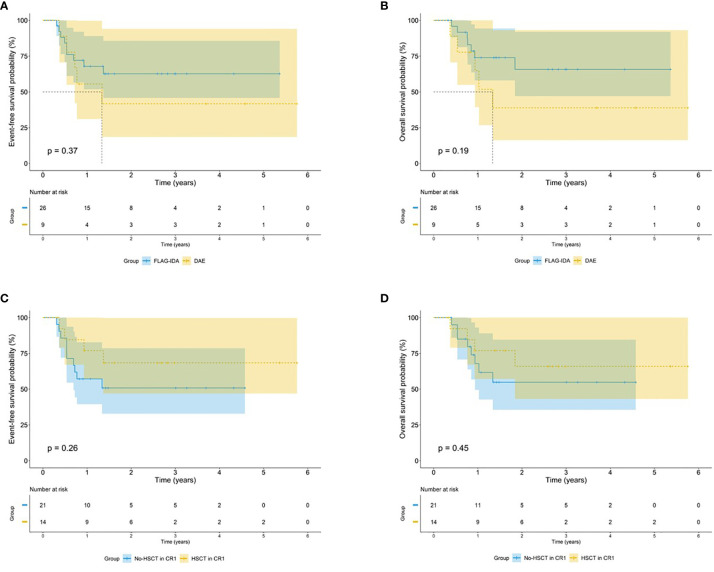
Survival outcomes by treatment regimens among *EVI1* ^high^ patients. Kaplan–Meier curve estimates the impact of different induction regimens on the **(A)** EFS and **(B)** OS in the cohort of *EVI1* ^high^ patients. Kaplan–Meier curve estimates the impact of HSCT after CR1 on the **(C)** EFS and **(D)** OS in the cohort of *EVI1* ^high^ patients.

Of the 35 patients with *EVI1* ^high^ who underwent an evaluation of the treatment response and survival data, the CR rate after the first course of chemotherapy of the 26 patients who received FLAG-IDA induction was significantly higher than that of the remaining 9 patients who received DAE induction (100% *vs.* 66%, *p* = 0.013). The Kaplan-Meier analysis showed that the FLAG-IDA group had a trend for a better 5-year EFS and OS, without a significant statistical difference (EFS: 62.7% *vs.* 41.7%, *p* = 0.37; OS: 65.7% *vs.* 38.9%, *p* = 0.19). A total of 14 patients with *EVI1* ^high^ who underwent HSCT after CR1 had a better 5-year EFS and OS, but this difference was not statistically different (EFS: 68.4% *vs.* 50.8%, *p* = 0.26; OS: 65.9% *vs.* 54.8%, *p* = 0.45) (**Figure 5**).

## 4 Discussion

In the present study, we retrospectively analyzed the characteristics and prognostic value of *EVI1* ^high^ in pediatric patients with AML. Our study enrolled pediatric patients aged 7–176 months in a large cohort of 421 pediatric patients with AML who received uniform treatments from multiple centers, with the inclusion of the respective FAB subtypes, cytogenetic characteristics, and molecular genetic characteristics, which are broadly representative. This study showed that *EVI1* ^high^ significantly correlated with unfavorable types, including MLL rearrangements, FAB-M7, and a complex karyotype, but was mutually exclusive for favorable types, including t (8;21), inv (16), an *NPM1-*mutation, and *CEBPA*-biallelic mutations. Furthermore, our results demonstrate that *EVI1* ^high^ is a poor independent prognostic factor for the survival in pediatric AML, especially in patients with *MLL* rearrangements. In view of its significant correlation with the survival, *EVI1* ^high^ is expected to be an excellent prognostic marker.

In this cohort, *EVI1* ^high^ was detected in 9.0% of cases, which was consistent with a previous pediatric study by Balgobind et al. (10), and several adult studies that indicated a percentage of 6%–11% (15, 16). However, the prevalence of *EVI1* ^high^ in our study was lower than the 28% reported by Ho et al. (9) (58/206) and 16% reported by Jo et al. (17) (21/130). This difference may reflect the distinct definitions in the studies, including the detection method, the cutoff value selection method, and the control gene used for normalization. In the study of Balgobind et al. (10), the cumulative relative expression of *EVI1-1A*, *-1B*, *and -3L* to a GAPDH above 1.5% is consistent with our definition of *EVI1* ^high^, showing the highest correlation with *EVI1* ^high^ cases based on the gene expression profiling; all normal bone marrow samples were below this threshold. In the study of Ho et al. (9), beta glucuronidase was quantified as an internal control, and *EVI1* ^high^ was defined as the cumulative relative expression of the total *EVI1 (*including *EVI1-1A*, *1B*, *1C*, *and 1D*), and the *MDS1* and *EVI1* complex fusion transcript, which was >1.0-fold increase compared to the normal peripheral blood controls. In the study of Jo et al. (17), patients with an *EVI1/ABL1* ratio higher than 0.1 were defined as *EVI1* ^high^. In our study, to avoid the inclusion of false-positive cases, patients were defined as *EVI1* ^high^ with an expression level of 10 or higher compared to a pool of 15 healthy bone marrow controls. The definition of *EVI1* ^high^ is still debatable, as the AML patient groups selected by the different definitions vary in size and may have an inferior prognosis. Thus, the studies may not be directly comparable, and standardization of *EVI1* transcript testing and reporting is required.

Previous studies have indicated that *EVI1* ^high^ is strongly associated with specific genetic and morphological subtypes in both pediatric and adult AML (9, 10, 15, 17), and the similarities and differences regarding *EVI1* ^high^ in pediatric vs. adult AML are shown in **Supplementary Table S4**. *EVI1* ^high^ in adult AML is frequently associated with and presumed to directly result from alterations in 3q26. In contrast, we detected only one patient with chromosomal rearrangements of 3q26 in our study. In pediatric AML, 3q26 abnormalities are rare. In the study of Balgobind et al., no 3q26 abnormalities were identified in pediatric AML patients with *EVI1* overexpression (10). A similar result was shown by the research of Jo et al., and the 3q26 abnormality was not detected either at the level of conventional cytogenetics or cryptically in the whole genome and transcriptome sequencing data (17). The mechanisms of *EVI1* overexpression in pediatric AML appear to be different from that of adult patients. We speculate that *EVI1* overexpression may not be the driving factor in pediatric AML, but a secondary event after leukemogenesis.

All patients with *EVI1* ^high^ in this study belonged to the intermediate- or high-risk cytogenetic/molecular aberrance group based on the current risk stratification (3, 18), as they showed *MLL* rearrangements and complex karyotypes. Furthermore, consistent with previous pediatric studies (9, 10, 17), *EVI1* ^high^ was also significantly associated with a FAB class M7 unrelated to trisomy 21, which has been reported to confer a poor prognosis in pediatric AML (19, 20), while *EVI1* ^high^ had a higher incidence of FAB-M5 in adult AML (21). Moreover, *EVI1* ^high^ was mutually exclusive with t (8;21), inv (16), *NPM1-*mutations, and *CEBPA*-biallelic mutations, representing favorable types of pediatric AML.

Although almost all relevant studies have shown an adverse impact of *EVI1* ^high^ on the therapeutic response and prognosis in adult AML (8, 15, 16), this was not completely consistent in pediatric AML. The reasons for these differences may lie with the therapy and prognosis between adults and children, but also in the different definitions of *EVI1* ^high^ and smaller sample sizes. In previous pediatric studies (9, 10, 17), *EVI1* ^high^ had significantly lower rates of EFS and OS but had no independent prognostic value for pediatric AML. In the present study, 421 pediatric patients with *de novo* AML were included, and patients with *EVI1* ^high^ had a significantly lower 5-year OS and EFS rates. Furthermore, *EVI1* ^high^ was also a significant predictor of a decreased OS and EFS in a separate univariate model and an independent prognostic factor for the OS but not EFS in a multivariate model including *EVI1* ^high^ and the aforementioned risk groups. To our knowledge, this study is the first to indicate that *EVI1* ^high^ has an independent prognostic value for pediatric AML.

*MLL* rearrangements are present in 15%–20% of pediatric patients with *de novo* AML and are associated with a poor prognosis (22). However, *MLL* rearrangements comprise a biologically and clinically heterogeneous group (23). A large international study of pediatric AML with *MLL* rearrangements identified specific translocations with prognostic associations (24). Previous studies have indicated that *EVI1* is a transcriptional target of *MLL* oncoproteins in hematopoietic stem cells and plays a critical role in tumor growth in a subset of *MLL*-r AML (25, 26). In a report of adult AML, *EVI1* ^high^ was the sole prognostic factor for the inferior OS, RFS, and EFS in both patients with *MLL*-r AML and patients with *MLL-AF9* (27). However, there have been inconsistent conclusions in the pediatric studies. Ho et al. (9) showed that *EVI1* ^high^ could not determine its prognostic value in pediatric AML with *MLL* rearrangements. Jo et al. (17) revealed that *EVI1* ^high^ was mainly detected and had a prognostic significance in myelomonocytic-lineage leukemia with *MLL* rearrangements. Matsuo et al. (28) also showed that *EVI1* ^high^ was an independent poor prognostic factor for the EFS but not OS in children with *MLL*-r AML. In the present study, *EVI1* ^high^ was associated with adverse EFS and OS in both the *MLL*-r and *MLL-AF9* subgroups. A multivariate analysis identified *EVI1* ^high^ as an independent prognostic factor predicting a poor EFS and OS in the total cohort of *MLL*-r AML, but not in the *MLL-AF9* subgroup, which may be caused by the smaller sample size.

At present, chemotherapy remains the front-line treatment for newly diagnosed pediatric AML, but the regimen for patients with *EVI1* ^high^ is not unified. Based on the medical research council AML15 trial (29), we have considered the FLAG-IDA or DAE regimen as induction chemotherapy. In the present study, the excellent CR rate of the FLAG-IDA regimen was significantly higher than that of the DAE regimen, which may indicate that the FLAG-IDA regimen is more suitable for induction in children with *EVI1* ^high^. Studies on whether pediatric AML patients with *EVI1* ^high^ need to undergo HSCT in CR1 are few. However, *EVI1^high^* is predominantly present in unfavorable cytogenetic subtypes (high or intermediate risk) and predicts adverse outcomes for all AML patients (also for the *MLL-r* subtype). Furthermore, previous research has shown that HSCT significantly improved the prognosis of adult AML patients with *EVI1* ^high^ and those with the *MLL*-r subtype as well (15, 27). Moreover, patients with *EVI1* ^high^ who underwent HSCT after CR1 had a higher OS and EFS than those who only received chemotherapy. Therefore, we suggest that pediatric AML patients (also in *MLL*-r subtype) with *EVI1^high^* should undergo HSCT in the first CR. However, it is still essential to conduct prospective multicenter clinical studies including more patients to confirm whether HSCT could improve the long-term survival of pediatric patients with AML and *EVI1* ^high^.

Moreover, novel therapeutic strategies effective for pediatric patients with *EVI1* ^high^ are also constantly being explored. Saito et al. (30) found that CD52 was highly expressed in most *EVI1* ^high^ leukemia cells, and humanized anti-CD52 monoclonal antibody CAMPATH-1H could inhibit cell growth and induce the apoptosis of *EVI1* ^high^ leukemia cells. These suggest that CAMPATH-1H may be effective in treating myeloid leukemia with *EVI1* ^high^. However, the correlation between CD52 and *EVI1* ^high^ in AML patients still needs to be verified because CD52 is not tested routinely during flow cytometry for establishing a diagnosis. Furthermore, whether CAMPATH-1H is effective in treating myeloid leukemia with *EVI1* ^high^ also needs to be clarified in clinical studies. Mittal et al. (31) reported that *EVI1*-induced hypermethylation and downregulation of miR-9 play an important role in leukemogenesis in pediatric patients with AML and *EVI1* ^high^, indicating that hypomethylating agents may be a potential therapeutic strategy for these patients. Nguyen et al. (32) showed that *EVI1* plays an important role in the key properties of AML leukemic stem cells, and all-trans retinoic acid (ATRA) enhances the effects of *EVI1* on AML stemness, thus, raising the possibility of using RAR antagonists in the therapy of *EVI1* ^high^ AML. However, Steinmetz et al. (33) and Verhagen et al. (34) demonstrated that primary AML cells with *EVI1* ^high^ were sensitive to ATRA, indicating that ATRA may be a candidate for patients with AML and *EVI1* ^high^. As noted above, *EVI1* ^high^ was associated with *MLL* rearrangement. As a highly effective inhibitor targeting MLL, Menin reduced leukemia burden significantly and prolonged survival in *in vivo* experiments, which indicated that Menin may be effective in treating myeloid leukemia with *EVI1* ^high^ (35, 36).

The limitation of this study is that it was a retrospective study and only 35 patients expressed *EVI1^high^*, and it will be necessary to explore more effective treatments through prospective multicenter studies to improve long-term outcomes of pediatric patients with AML and *EVI1* ^high^.

In conclusion, *EVI1* ^high^ is significantly associated with specific unfavorable cytogenetic (*MLL* rearrangements and complex karyotypes) and morphologic (FAB-M7) subtypes in pediatric AML. Furthermore, this study is the first to indicate that *EVI1* ^high^ is an independent adverse prognosis predictor for survival, especially in *MLL*-r AML. These results may be conducive to risk stratification and therapy decisions in pediatric patients with AML, and *EVI1* transcript levels should be routinely assessed at diagnosis for risk stratification once a standard laboratory protocol is established and the cutoff value is determined.

## Data Availability Statement

The original contributions presented in the study are included in the article/**Supplementary Material**. Further inquiries can be directed to the corresponding authors.

## Ethics Statement

The studies involving human participants were reviewed and approved by the ethics committees from Fujian Medical University Union Hospital, Nanfang Hospital, TaiXin Hospital, Hunan Children’s Hospital, Sun Yat-sen Memorial Hospital, Guangzhou Women and Children’s Medical Center, and People’s Hospital of Hunan Province. Written informed consent to participate in this study was provided by the participants’ legal guardian/next of kin.

## Author Contributions

YZ, JL, and JH conceived and designed the experiments. YZ performed the experiments. YZ, SL, HZ, XHu, ZC, XF, CL, MZ, HX, YHe, and XHe collected the clinical data. YZ and YHu analyzed and interpreted the data. YZ and YHu wrote the manuscript. JH critically revised the manuscript. All authors contributed to the article and approved the submitted version.

## Funding

This work was supported by the Construction Project of the Fujian Medical Center of Hematology (Min201704), Startup fund of scientific research, Fujian medical university (2019QH1022, 2019QH1032).

## Conflict of Interest

The authors declare that the research was conducted in the absence of any commercial or financial relationships that could be construed as a potential conflict of interest.

## Publisher’s Note

All claims expressed in this article are solely those of the authors and do not necessarily represent those of their affiliated organizations, or those of the publisher, the editors and the reviewers. Any product that may be evaluated in this article, or claim that may be made by its manufacturer, is not guaranteed or endorsed by the publisher.

## Supplementary Material

Supplementary Figure 1 C-HUANAN-AML15 protocol workflow. FLAG-IDA: Fludarabine 30 mg/m2/d d2-6, cytarabine 2g/m2/d d2-6, Idarubicin 8 mg/m2/d d4-6, Granulocyte Colony Stimulating Factor (G-CSF) 5μg/kg/d d1-7. DAE (3 + 10+5): Daunorubicin 50 mg/m2/d d1,3,5; Cytarabine 100mg/m2 q12h d1-10, Etoposide 100mg/m2/d d1-5. DAE (3 + 8+5): Daunorubicin 50 mg/m2/d d1,3,5; Cytarabine 100mg/m2 q12h d1-8, Etoposide 100mg/m2/d d1-5. HAE: Homoharringtonine 3mg/m2/d d1-5, Cytarabine 100mg/m2 q12h d1-7, Etoposide 100mg/m2/d d1-5. HHA: Homoharringtonine 3mg/m2/d d1-7, Cytarabine 2g/m2 q12h d1-3. MidAc: Mitoxantrone 10mg/m2/d d1-5, Cytarabine 1g/m2 q12h d1-3.

## References

[1] Kolb EA Meshinchi S. Acute Myeloid Leukemia in Children and Adolescents: Identification of New Molecular Targets Brings Promise of New Therapies. Hematol Am Soc Hematol Educ Program (2015) 2015(1):507–13. doi: 10.1182/asheducation-2015.1.507 26637766

[2] Taga T Tomizawa D Takahashi H Adachi S. Acute Myeloid Leukemia in Children: Current Status and Future Directions. Pediatr Int (2016) 2:71–80. doi: 10.1111/ped.12865 26645706

[3] Döhner H Estey E Grimwade D Amadori S Appelbaum FR Büchner T. Diagnosis and Management of AML in Adults: 2017 ELN Recommendations From an International Expert Panel. Blood (2017) 4:424–47. doi: 10.1182/blood-2016-08-733196 PMC5291965 27895058

[4] Mawad R Estey EH. Acute Myeloid Leukemia With Normal Cytogenetics. Curr Oncol Rep (2012) 5:359–68. doi: 10.1007/s11912-012-0252-x 22806102

[5] Qin YZ Zhao T Zhu HH Wang J Jia JS Lai YY. High EVI1 Expression Predicts Poor Outcomes in Adult Acute Myeloid Leukemia Patients With Intermediate Cytogenetic Risk Receiving Chemotherapy. Med Sci Monit (2018) 24:758–67. doi: 10.12659/msm.905903 PMC5810369 29408852

[6] Kataoka K Kurokawa M. Ecotropic Viral Integration Site 1, Stem Cell Self-Renewal and Leukemogenesis. Cancer Sci (2012) 8:1371–7. doi: 10.1111/j.1349-7006.2012.02303.x PMC7659387 22494115

[7] Goyama S Kurokawa M. Pathogenetic Significance of Ecotropic Viral Integration Site-1 in Hematological Malignancies. Cancer Sci (2009) 6:990–5. doi: 10.1111/j.1349-7006.2009.01152.x PMC11158526 19385966

[8] Hinai AA Valk PJ. Review: Aberrant EVI1 Expression in Acute Myeloid Leukaemia. Br J Haematol (2016) 6:870–8. doi: 10.1111/bjh.13898 26729571

[9] Ho PA Alonzo TA Gerbing RB Pollard JA Hirsch B Raimondi SC. High EVI1 Expression is Associated With MLL Rearrangements and Predicts Decreased Survival in Paediatric Acute Myeloid Leukaemia: A Report From the Children's Oncology Group. Br J Haematol (2013) 5:670–7. doi: 10.1111/bjh.12444 PMC3754879 23826732

[10] Balgobind BV Lugthart S Hollink IH Arentsen-Peters ST van Wering ER de Graaf SS. EVI1 Overexpression in Distinct Subtypes of Pediatric Acute Myeloid Leukemia. Leukemia (2010) 5:942–9. doi: 10.1038/leu.2010.47 20357826

[11] Arber DA Orazi A Hasserjian R Thiele J Borowitz MJ Le Beau MM. The 2016 Revision to the World Health Organization Classification of Myeloid Neoplasms and Acute Leukemia. Blood (2016) 20:2391–405. doi: 10.1182/blood-2016-03-643544 27069254

[12] Beillard E Pallisgaard N van der Velden VH Bi W Dee R van der Schoot E. Evaluation of Candidate Control Genes for Diagnosis and Residual Disease Detection in Leukemic Patients Using 'Real-Time' Quantitative Reverse-Transcriptase Polymerase Chain Reaction (RQ-PCR) - a Europe Against Cancer Program. Leukemia (2003) 12:2474–86. doi: 10.1038/sj.leu.2403136 14562124

[13] Schmittgen TD Livak KJ. Analyzing Real-Time PCR Data by the Comparative C(T) Method. Nat Protoc (2008) 6:1101–8. doi: 10.1038/nprot.2008.73 18546601

[14] Santamaría CM Chillón MC García-Sanz R Pérez C Caballero MD Ramos F. Molecular Stratification Model for Prognosis in Cytogenetically Normal Acute Myeloid Leukemia. Blood (2009) 1:148–52. doi: 10.1182/blood-2008-11-187724 19398719

[15] Gröschel S Lugthart S Schlenk RF Valk PJ Eiwen K Goudswaard C. High EVI1 Expression Predicts Outcome in Younger Adult Patients With Acute Myeloid Leukemia and is Associated With Distinct Cytogenetic Abnormalities. J Clin Oncol (2010) 12:2101–7. doi: 10.1200/jco.2009.26.0646 20308656

[16] Lugthart S van Drunen E van Norden Y van Hoven A Erpelinck CA Valk PJ. High EVI1 Levels Predict Adverse Outcome in Acute Myeloid Leukemia: Prevalence of EVI1 Overexpression and Chromosome 3q26 Abnormalities Underestimated. Blood (2008) 8:4329–37. doi: 10.1182/blood-2007-10-119230 18272813

[17] Jo A Mitani S Shiba N Hayashi Y Hara Y Takahashi H. High Expression of EVI1 and MEL1 is a Compelling Poor Prognostic Marker of Pediatric AML. Leukemia (2015) 5:1076–83. doi: 10.1038/leu.2015.5 25567132

[18] Rau RE Loh ML. Using Genomics to Define Pediatric Blood Cancers and Inform Practice. Hematol Am Soc Hematol Educ Program (2018) 1:286–300. doi: 10.1182/asheducation-2018.1.286 PMC6245969 30504323

[19] Teyssier AC Lapillonne H Pasquet M Ballerini P Baruchel A Ducassou S. Acute Megakaryoblastic Leukemia (Excluding Down Syndrome) Remains an Acute Myeloid Subgroup With Inferior Outcome in the French ELAM02 Trial. Pediatr Hematol Oncol (2017) 8:425–7. doi: 10.1080/08880018.2017.1414905 29303660

[20] Schweitzer J Zimmermann M Rasche M von Neuhoff C Creutzig U Dworzak M. Improved Outcome of Pediatric Patients With Acute Megakaryoblastic Leukemia in the AML-BFM 04 Trial. Ann Hematol (2015) 8:1327–36. doi: 10.1007/s00277-015-2383-2 PMC4488462 25913479

[21] He X Wang Q Cen J Qiu H Sun A Chen S. Predictive Value of High EVI1 Expression in AML Patients Undergoing Myeloablative Allogeneic Hematopoietic Stem Cell Transplantation in First CR. Bone Marrow Transplant (2016) 7:921–7. doi: 10.1038/bmt.2016.71 27042849

[22] Conneely S Rau R. The Genomics of Acute Myeloid Leukemia in Children. Cancer Metastasis Rev (2020) 1:189–209. doi: 10.1007/s10555-020-09846-1 PMC7324027 31925603

[23] Winters A Bernt K. MLL-Rearranged Leukemias-An Update on Science and Clinical Approaches. Front Pediatr (2017) 4:4. doi: 10.3389/fped.2017.00004 PMC5299633 28232907

[24] Balgobind BV Raimondi SC Harbott J Zimmermann M Alonzo TA Auvrignon A. Novel Prognostic Subgroups in Childhood 11q23/MLL-Rearranged Acute Myeloid Leukemia: Results of an International Retrospective Study. Blood (2009) 12:2489–96. doi: 10.1182/blood-2009-04-215152 PMC2927031 19528532

[25] Arai S Yoshimi A Shimabe M Ichikawa M Nakagawa M Imai Y. Evi-1 Is a Transcriptional Target of Mixed-Lineage Leukemia Oncoproteins in Hematopoietic Stem Cells. Blood (2011) 23:6304–14. doi: 10.1182/blood-2009-07-234310 21190993

[26] Bindels EM Havermans M Lugthart S Erpelinck C Wocjtowicz E Krivtsov AV. EVI1 is Critical for the Pathogenesis of a Subset of MLL-AF9-Rearranged AMLs. Blood (2012) 24:5838–49. doi: 10.1182/blood-2011-11-393827 PMC3382941 22553314

[27] Gröschel S Schlenk RF Engelmann J Rockova V Teleanu V Kühn MW. Deregulated Expression of EVI1 Defines a Poor Prognostic Subset of MLL-Rearranged Acute Myeloid Leukemias: A Study of the German-Austrian Acute Myeloid Leukemia Study Group and the Dutch-Belgian-Swiss HOVON/SAKK Cooperative Group. J Clin Oncol (2013) 1:95–103. doi: 10.1200/jco.2011.41.5505 23008312

[28] Matsuo H Kajihara M Tomizawa D Watanabe T Saito AM Fujimoto J. EVI1 Overexpression is a Poor Prognostic Factor in Pediatric Patients With Mixed Lineage Leukemia-AF9 Rearranged Acute Myeloid Leukemia. Haematologica (2014) 11:e225–7. doi: 10.3324/haematol.2014.107128 PMC4222468 25015941

[29] Burnett AK Russell NH Hills RK Hunter AE Kjeldsen L Yin J. Optimization of Chemotherapy for Younger Patients With Acute Myeloid Leukemia: Results of the Medical Research Council AML15 Trial. J Clin Oncol (2013) 27:3360–8. doi: 10.1200/jco.2012.47.4874 23940227

[30] Saito Y Nakahata S Yamakawa N Kaneda K Ichihara E Suekane A. CD52 as a Molecular Target for Immunotherapy to Treat Acute Myeloid Leukemia With High EVI1 Expression. Leukemia (2011) 6:921–31. doi: 10.1038/leu.2011.36 21394097

[31] Mittal N Li L Sheng Y Hu C Li F Zhu T. A Critical Role of Epigenetic Inactivation of miR-9 in EVI1(high) Pediatric AML. Mol Cancer (2019) 1:30. doi: 10.1186/s12943-019-0952-z PMC6391809 30813931

[32] Nguyen CH Bauer K Hackl H Schlerka A Koller E Hladik A. All-Trans Retinoic Acid Enhances, and a Pan-RAR Antagonist Counteracts, the Stem Cell Promoting Activity of EVI1 in Acute Myeloid Leukemia. Cell Death Dis (2019) 12:944. doi: 10.1038/s41419-019-2172-2 PMC6904467 31822659

[33] Steinmetz B Hackl H Slabáková E Schwarzinger I Smějová M Spittler A. The Oncogene EVI1 Enhances Transcriptional and Biological Responses of Human Myeloid Cells to All-Trans Retinoic Acid. Cell Cycle (2014) 18:2931–43. doi: 10.4161/15384101.2014.946869 PMC4613657 25486480

[34] Verhagen HJ Smit MA Rutten A Denkers F Poddighe PJ Merle PA. Primary Acute Myeloid Leukemia Cells With Overexpression of EVI-1 are Sensitive to All-Trans Retinoic Acid. Blood (2016) 4:458–63. doi: 10.1182/blood-2015-07-653840 26582376

[35] Dzama MM Steiner M Rausch J Sasca D Schönfeld J Kunz K. Synergistic Targeting of FLT3 Mutations in AML via Combined Menin-MLL and FLT3 Inhibition. Blood (2020) 21:2442–56. doi: 10.1182/blood.2020005037 PMC8215191 32589720

[36] Krivtsov AV Evans K Gadrey JY Eschle BK Hatton C Uckelmann HJ. A Menin-MLL Inhibitor Induces Specific Chromatin Changes and Eradicates Disease in Models of MLL-Rearranged Leukemia. Cancer Cell (2019) 6:660–73.e11. doi: 10.1016/j.ccell.2019.11.001 PMC7227117 31821784

