# Supplementary material for: High EVI1 Expression Predicts Adverse Outcomes in Children With De Novo Acute Myeloid Leukemia
Source: Front Oncol. 2021 Sep 13;11:712747. doi: 10.3389/fonc.2021.712747 (PMC8474639; doi:10.3389/fonc.2021.712747)
Supplement: Supplementary Figure 1 — C-HUANAN-AML15 protocol workflow. FLAG-IDA: Fludarabine 30 mg/m2/d d2-6, cytarabine 2g/m2/d d2-6, Idarubicin 8 mg/m2/d d4-6, Granulocyte Colony Stimulating Factor (G-CSF) 5μg/kg/d d1-7. DAE (3 + 10+5): Daunorubicin 50 mg/m2/d d1,3,5; Cytarabine 100mg/m2 q12h d1-10, Etoposide 100mg/m2/d d1-5. DAE (3 + 8+5): Daunorubicin 50 mg/m2/d d1,3,5; Cytarabine 100mg/m2 q12h d1-8, Etoposide 100mg/m2/d d1-5. HAE: Homoharringtonine 3mg/m2/d d1-5, Cytarabine 100mg/m2 q12h d1-7, Etoposide 100mg/m2/d d1-5. HHA: Homoharringtonine 3mg/m2/d d1-7, Cytarabine 2g/m2 q12h d1-3. MidAc: Mitoxantrone 10mg/m2/d d1-5, Cytarabine 1g/m2 q12h d1-3. [file DataSheet_1.docx]

**Table S1. Prognostic risk systems in C-HUANAN-AML15 protocol**

| Risk stratification | Genetic abnormality and induction chemotherapy response |
| --- | --- |
| Low risk criteria | Include one of the f0llowing genetic abnormality and CR after induction 1: t(8;21)(q22;q22)；AML/ETO（RUNX1- RUNX1T1）;  inv(16)(p13q22)/t(16;16)(p13;q22)；CBFB-MYH11；  normal cytogenetics: NPM1 or isolated biallelic (double) CEBPA mutation in the absence of FLT3-ITD |
| Intermediate risk criteria | Exclude low risk or high risk genetic abnormality and blast in bone＜15% after induction1 and CR after induction2 |
| High risk criteria | Include one of the f0llowing genetic abnormal blast in bone≥15% after induction1 or no CR after induction2:  Mutated FLT3-ITD;  Complex karyotype;  -5 or del(5q);  abn(3q);  abn(17p);  -7 or del(7q) |

**
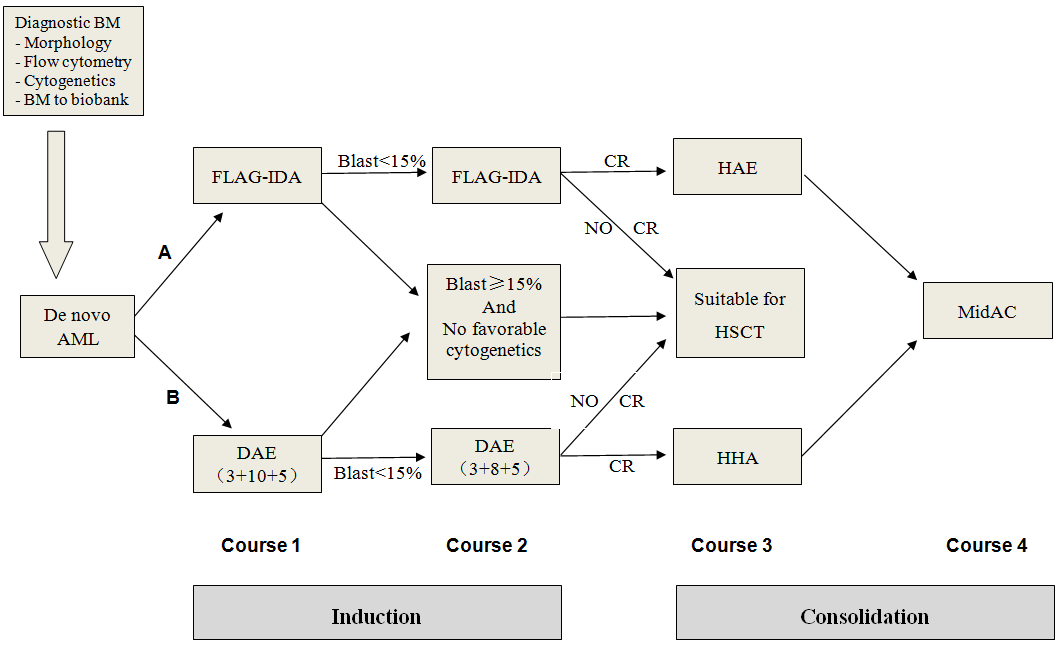
**

**Figure S1 C-HUANAN-AML15 protocol workflow. FLAG-IDA: Fludarabine 30 mg/m^2^/d d2-6, cytarabine 2g/ m^2^/d d2-6, Idarubicin 8 mg/m^2^/d d4-6, Granulocyte Colony Stimulating Factor (G-CSF) 5μg/kg/d d1-7. DAE (3+10+5): Daunorubicin 50 mg/m^2^/d d1,3,5; Cytarabine 100mg/m^2^ q12h d1-10, Etoposide 100mg/m^2^/d d1-5. DAE (3+8+5): Daunorubicin 50 mg/m^2^/d d1,3,5; Cytarabine 100mg/m^2^ q12h d1-8, Etoposide 100mg/m^2^/d d1-5. HAE: Homoharringtonine 3mg/m^2^/d d1-5, Cytarabine 100mg/m^2^ q12h d1-7, Etoposide 100mg/m^2^/d d1-5. HHA: Homoharringtonine 3mg/m^2^/d d1-7, Cytarabine 2g/m^2^ q12h d1-3. MidAc: Mitoxantrone 10mg/m^2^/d d1-5, Cytarabine 1g/m^2^ q12h d1-3.**

**Table S2. Clinical and Genetic Characteristics According to EVI1 Status in patients with MLL rearrangement.**

| Characteristic | MLL patients (n=70) | EVI1^high^ group (n=15) | EVI1^low^ group (n=55) | *P* |
| --- | --- | --- | --- | --- |
| Age, months |  |  |  | 0.584 |
| Median (range) | 44.5(8-168) | 60(12-163) | 42(8-168) |  |
| Sex, n% |  |  |  | 1.000 |
| Male | 41(58.6) | 9(60.0) | 32(58.2) |  |
| Female | 29(41.4) | 6(40.0) | 23(41.8) |  |
| WBC, ×10^9^/L |  |  |  | 1.000 |
| <50 | 47(67.1) | 10(66.7) | 37(67.3) |  |
| ≥50 | 23(32.9) | 5(33.3) | 18(32.7) |  |
| FAB subtype, n% |  |  |  | 1.000 |
| M7 | 4(5.7) | 1(6.7) | 3(5.5) |  |
| Other types | 66(94.3) | 14(93.3) | 52(94.5) |  |
| *Cytogenetic characteristics, n(%) |  |  |  |  |
| -7 or del(7q) | 1(1.4) | 1(6.7) | 0(0) | 0.214 |
| Complex karyotype | 2(2.9) | 1(6.7) | 1(1.8) | 1.000 |
| †Cytogenetic risk |  |  |  | 0.020 |
| Intermediate | 38(54.3) | 4(26.7) | 34(61.8) |  |
| Unfavorable | 32(45.6) | 11(73.3) | 21(38.2) |  |
| Molecular abnormalities |  |  |  |  |
| FLT3-ITD | 5(7.1) | 2(13.3) | 5(5.5) | 0.577 |
| ASXL1 | 8(11.4) | 0(0) | 8(14.5) | 0.187 |
| CEBPA- mutation | 1(1.4) | 0(0) | 1(1.8) | 1.000 |
| CR after induction 2^nd^ |  |  |  | 0.348 |
| Yes | 60(85.7) | 11(73.3) | 49(89.1) |  |
| No | 5(7.1) | 2(13.3) | 3(5.5) |  |
| Missing | 5(7.1) | 2(13.3) | 3(5.5) |  |
| Blast>15% in BM after induction 1^st^ |  |  |  | 0.494 |
| Yes | 5(7.1) | 2(13.3) | 3(5.5) |  |
| No | 64(91.4) | 13(86.7) | 51(92.7) |  |
| Missing | 1(1.4) | 0(0) | 1(1.8) |  |

***** **Patients may be counted more than once owing to the coexistence of more than one cytogenetic abnormality in the leukemic clone.**

**†Favorable risk: t(15;17), t(8;21), inv(16)/t(16;16); unfavorable risk: inv(3) or t(3;3), t(6;9), t(v;11q23) other than t(9;11), -5 or del(5q), -7 or del(7q), abn(17p), complex karyotype (three or more abnormalities in the absence of a WHO designated recurring chromosome abnormality); intermediate risk: all chromosome abnormalities not classified as favorable or unfavorable. ^#^Only 383 patients were included in this part, for 38 cases giving up treatment or loss to follow-up.**

**Table S3. Univariate and multivariate analysis of patients with MLL-AF9 rearrangement.**

| Cases (n=29) | EFS | | | | OS | | | | |  |
| --- | --- | --- | --- | --- | --- | --- | --- | --- | --- | --- |
| **Univariate Analysis** | **HR** | **95% CI** | ***P* value** | | **HR** | **95% CI** | | ***P* value** | |  |
| Age (+1 year) | 0.988 | 0.960-1.016 | | 0.396 | 0.993 | | 0.965-1.020 | | 0.593 | |
| Gender (Male) | 3.386 | 0.376-30.456 | | 0.277 | 2.516 | | 0.260-24.359 | | 0.426 | |
| WBC (≥50X10^9^/L) | 4.080 | 0.678-24.572 | | 0.125 | 5.464 | | 0.768-38.886 | | 0.090 | |
| FAB (M7) | 2.300 | 0.254-20.813 | | 0.458 | 2.989 | | 0.303-29.520 | | 0.349 | |
| Risk Category1^*^ | 2.526 | 0.279-22.835 | | 0.409 | 2.883 | | 0.299-27.770 | | 0.360 | |
| Risk Category2^@^ | 4.635 | 0.774-27.761 | | 0.093 | 8.484 | | 0.882-81.632 | | 0.064 | |
| EVI1^high^ | 7.112 | 1.182-42.794 | | 0.032 | 13.349 | | 1.384-128.742 | | 0.025 | |
| ASXL1 mutation | 0.034 | 0.000-372.842 | | 0.477 | 0.035 | | 0.000-1310.839 | | 0.533 | |
| Induction protocol (DAE) | 14.337 | 1.598-128.614 | | 0.017 | 10.209 | | 1.061-98.231 | | 0.044 | |
| No CR after 2^nd^ course | 25.456 | 1.590-407.465 | | 0.022 | 25.456 | | 1.590-407.465 | | 0.022 | |
| Blast>15% in BM after 1st course | 4.061 | 0.449-36.725 | | 0.212 | 5.179 | | 0.533-50.307 | | 0.156 | |
| **Multivariate Analysis** | **HR** | **95% CI** | ***P* value** | | **HR** | **95% CI** | | ***P* value** | |  |
| EVI1^high^ | 10.091 | 0.858-118.713 | 0.066 | | 13.056 | 0.901-189.160 | | 0.060 | |  |
| Induction protocol (DAE) | 18.317 | 1.370-244.949 | 0.028 | | 9.792 | 0.676-141.870 | | 0.094 | |  |
| No CR after 2nd course | 2.092 | 0.115-38.204 | 0.618 | | 2.188 | 0.117-40.812 | | 0.600 | |  |

*Risk category based on treatment regimens. Refer to Supplementary TableS1.

@ Risk category based on cytogenetic stratification of ELN 2017.

**Table S4. The similarities and differences regarding *EVI1*^high^ in pediatric *vs* adult AML**

|  | Pediatric AML | | Adult AML |
| --- | --- | --- | --- |
| Incidence (%) | | 9~28(1-3) | 7.8~15.4%(4, 5) |
| Associated with FAB type | | M7(1), M4/5/7(2), M6/7(3) | M5(6) |
| Associated with 3q26 abnormalities | | no 3q26 abnormalities were identified in *EVI1^high^* pediatric AML(1-3), 2.6% (1/38) 3q26 abnormality was identified in our study. | *EVI1^high^* was found in 65% 3q AML and 45% 3q26 AML(5); 3q26 was significant higher in *EVI^high^* AML than *EVI^low^* AML (15.4% vs 0.2%, *P*<0.001)(4). |
| Associated with *MLL-r* | | *EVI1^high^* was detected in 27.7%~36% AML patients with *MLL-r* and 37.9% in patients with *MLL-AF9* (3, 7); and *MLL-r* occurred in 40% of *EVI1^high^* patients as opposed to 12% of the *EVI1^low^* patients (*P*<0.001)(1). | *EVI1^high^* was found in 45.8% of all patients with *MLL-r*, with *MLL-AF6* showing the highest frequency (83.9%), followed by *MLL-AF9* at 40.0%(8); and *MLL-r* occurred in 13.2% of *EVI1^high^* patients as opposed to 1.3% of the *EVI1^low^* patients (*P*<0.001)(4). |
| Associated with other cytogenetic Abnormalities | | *EVI1^high^* was virtually absent in favourable-risk AML, including core-binding factor AML, t(15;17), double mutations in the myeloid transcription factor gene *CEBPA* or mutations in nucleophosmin (*NPM1*) without concurrent mutation in the haematopoietic receptor *FLT3*, but significantly associated with monosomy 7(3-5). | |
| Prognosis value | | *EVI1^high^* patients had significantly lower EFS and OS. However, in multivariate analysis including other established prognostic markers, *EVI1* expression did not retain independent prognostic significance(1, 3). | *EVI1^high^* has been shown to act as an independent adverse prognostic marker for complete remission (CR), overall- (OS), relapse free- (RFS) and event-free (EFS) survival in AML, i.e., irrespective of the presence of 3q26 rearrangements(4, 5). Moreover, *EVI1^high^* defines poor prognostic subsets among AML with *MLL-r* and AML with *MLL-AF9*(8). |
| Allogeneic hematopoietic stem cell transplantation (all-HSCT) | | There was no aveilable study in literature about whether HSCT could improve suvival outcome of pediatric AML patients with *EVI1^high^*. In our study, patients with *EVI1^high^* who underwent allo-HSCT after CR1 had higher OS and EFS than those who only received chemotherapy, but the difference was not statistically significant. (EFS: 68.4% vs. 50.8%, *p* = 0.26; OS: 65.9% vs. 54.8%, *p* = 0.45) | Patients with *EVI1^high^* AML, especially in *MLL-r* subtype seem to benefit from allo-HSCT in first CR(4, 8). |

References

1. Ho PA, Alonzo TA, Gerbing RB, Pollard JA, Hirsch B, Raimondi SC, et al. High EVI1 expression is associated with MLL rearrangements and predicts decreased survival in paediatric acute myeloid leukaemia: a report from the children's oncology group. *Br J Haematol*.(2013) 5: 670-7. doi:10.1111/bjh.12444

2. Jo A, Mitani S, Shiba N, Hayashi Y, Hara Y, Takahashi H, et al. High expression of EVI1 and MEL1 is a compelling poor prognostic marker of pediatric AML. *Leukemia*.(2015) 5: 1076-83. doi:10.1038/leu.2015.5

3. Balgobind BV, Lugthart S, Hollink IH, Arentsen-Peters ST, van Wering ER, de Graaf SS, et al. EVI1 overexpression in distinct subtypes of pediatric acute myeloid leukemia. *Leukemia*.(2010) 5: 942-9. doi:10.1038/leu.2010.47

4. Gröschel S, Lugthart S, Schlenk RF, Valk PJ, Eiwen K, Goudswaard C, et al. High EVI1 expression predicts outcome in younger adult patients with acute myeloid leukemia and is associated with distinct cytogenetic abnormalities. *J Clin Oncol*.(2010) 12: 2101-7. doi:10.1200/jco.2009.26.0646

5. Hinai AA, Valk PJ. Review: Aberrant EVI1 expression in acute myeloid leukaemia. *Br J Haematol*.(2016) 6: 870-8. doi:10.1111/bjh.13898

6. He X, Wang Q, Cen J, Qiu H, Sun A, Chen S, et al. Predictive value of high EVI1 expression in AML patients undergoing myeloablative allogeneic hematopoietic stem cell transplantation in first CR. *Bone Marrow Transplant*.(2016) 7: 921-7. doi:10.1038/bmt.2016.71

7. Matsuo H, Kajihara M, Tomizawa D, Watanabe T, Saito AM, Fujimoto J, et al. EVI1 overexpression is a poor prognostic factor in pediatric patients with mixed lineage leukemia-AF9 rearranged acute myeloid leukemia. *Haematologica*.(2014) 11: e225-7. doi:10.3324/haematol.2014.107128

8. Gröschel S, Schlenk RF, Engelmann J, Rockova V, Teleanu V, Kühn MW, et al. Deregulated expression of EVI1 defines a poor prognostic subset of MLL-rearranged acute myeloid leukemias: a study of the German-Austrian Acute Myeloid Leukemia Study Group and the Dutch-Belgian-Swiss HOVON/SAKK Cooperative Group. *J Clin Oncol*.(2013) 1: 95-103. doi:10.1200/jco.2011.41.5505
